# A Case of Recovery From COVID-19 Pneumonia in Pregnancy After Vaginal Delivery in ICU

**DOI:** 10.7759/cureus.19862

**Published:** 2021-11-24

**Authors:** Maryellen Campbell, Sai Palati, Anthony Sanchez, Martin Castaneda

**Affiliations:** 1 Obstetrics and Gynecology, Charles E. Schmidt College of Medicine, Florida Atlantic University, Boca Raton, USA; 2 Obstetrics and Gynecology, Bethesda Hospital East, Boynton Beach, USA

**Keywords:** sars-cov-2 (severe acute respiratory syndrome corona virus 2), critical care in obstetrics, covid-19 pneumonia, ground-glass opacity, spontaneous vaginal delivery, intensive respiratory care, vacuum-assisted

## Abstract

The severe acute respiratory syndrome coronavirus 2 (SARS-CoV-2) has important implications for gravid patients as they are more likely to experience severe complications of pregnancy such as multisystem inflammatory syndrome if infected with coronavirus disease 2019 (COVID-19). Due to normal physiological adaptations of pregnancy, COVID-19 may strain an already stressed respiratory system, making delivery a viable treatment option. We present a case of a gravid patient infected with COVID-19 pneumonia who delivered vaginally in the intensive care unit (ICU) at our hospital. Further research into clinical progress and management of pregnancy complicated by COVID-19 is necessary.

## Introduction

The severe acute respiratory syndrome coronavirus 2 (SARS-CoV-2) responsible for coronavirus disease 2019 (COVID-19), along with other coronaviruses, can cause a spectrum of disease manifestations ranging from the common cold to pneumonia to severe acute respiratory syndrome (SARS). This disease has important implications for gravid patients [1-4]. First, pregnancy is potentially a risk factor for COVID-19 infection, as dampened immune function and the expression of angiotensin-converting enzyme 2 (ACE2) receptors in trophoblastic tissues put pregnant women at a greater risk for contracting the disease [5]. In fact, inflammatory changes in the placenta including thrombotic and vascular abnormalities have been observed in the postpartum analysis of infected patients [6]. Pregnant women are at greater risk for developing pneumonia presenting with fever, cough, and dyspnea when infected with COVID-19 [2]. For coronavirus infections resulting in SARS, an elevated proportion of unfavorable outcomes including preterm birth (<37 weeks), premature prelabor rupture of membranes (PPROM), preeclampsia, and fetal growth restriction (FGR) have been reported [2]. Another important association of SARS complicated coronavirus infection is neonatal intensive care unit (NICU) admission and perinatal death [2]. Women with preexisting medical conditions such as hypertension, diabetes, and obesity are at higher risk of adverse events [7]. Obstetric complications are not the only concern for expecting mothers. Pregnant patients are more likely to experience severe complications such as multisystem inflammatory syndrome involving myocarditis when also suffering from COVID-19 [1]. Additionally, the INTERCOVID Multinational Cohort Study has shown that infected women may have a mortality risk that is 22 times greater than uninfected pregnant women [7].

## Case presentation

A 34-year-old female G2P0010 at 33 4/7 weeks of gestation presented to the emergency department with a chief complaint of fever, chills, runny nose, and watery eyes for four days and subsequently tested positive for COVID-19. She denied receiving the COVID-19 vaccine. The patient denied sick contacts as she was only exposed to her live-in partner who was asymptomatic at the time. She denied cough, shortness of breath, chest pain, nausea, vomiting, diarrhea, constipation, contractions, leakage of fluid, and vaginal bleeding. She endorsed good fetal movement. Her past medical history was notable for uterine fibroids, but her family medical history was non-contributory. Her only medication was prenatal vitamins. Her physical examination was within normal limits, and initial lab values were significant for transaminitis, with aspartate aminotransferase (AST) and alanine aminotransferase (ALT) three to four times the upper limit of normal, believed to be COVID-related.

She was admitted to the labor and delivery floor for observation and supportive treatment where she spiked fevers but remained hemodynamically stable for four days. Fetal assessment was reassuring with reactive nonstress test (NST) and normal biophysical profile (BPP), performed daily. She required O_2_ supplementation and O_2_ requirements steadily increased as her respiratory status worsened. On day four of inpatient management, she received a COVID-19 convalescent plasma (CCP) infusion. On day five, she developed shortness of breath, tachypnea, and desaturated to 84% oxygen on room air. The patient developed a compensated respiratory alkalosis and was transferred to the ICU where she received 5 L O_2_ via high-flow nasal cannula. She started a five-day course of remdesivir and a 10-day course of dexamethasone.

On day nine in the ICU at 34 6/7 weeks of gestation, she was induced for maternal benefit with Cervidil (dinoprostone) 10 mg and Cook cervical ripening balloon (Bloomington, IN: Cook Medical) due to worsening respiratory status. After artificial membrane rupture, we performed a shortened second stage of labor with vacuum-assisted vaginal delivery in the ICU. The appearance, pulse, grimace, activity, and respiration (APGAR) scores were nine and nine at one and five minutes, respectively, and the baby weighed 4 pounds 14 ounces. The estimated blood loss was 200 mL and a second-degree laceration was repaired. Neonate was transferred to the newborn intensive care unit (NICU) and underwent routine preterm neonate testing and care. The patient tolerated delivery well. She received routine postpartum care as her pain was controlled, she ambulated and tolerated oral fluids and nutrition, and passed flatus. She continued to be managed in the ICU from a respiratory standpoint.

Her respiratory status steadily improved, and she was transferred to the floor on 2 L O_2_ nasal cannula (NC) on postpartum day seven with O_2_ saturation between 96% and 98%. Her transaminitis resolved. On postpartum day 11, 22 days after initially presenting to the emergency department, she was discharged home with oxygen supplementation.

## Discussion

Pregnancy is associated with respiratory and immunological adaptations as the fetus develops. Total body oxygen consumption increases by approximately 20% as compared to non-pregnant levels with a significant increase in minute ventilation and resulting in compensated respiratory alkalosis [8]. Pregnant patients may experience physiologic dyspnea as well but should still be evaluated for possible respiratory or cardiac illness at presentation [8]. An important example of immunologic adaptation in pregnancy that has implications in respiratory compromise is the attenuation of adaptive immune function, most notable during the third trimester [9].

COVID-19 is known to target the respiratory system, and respiratory distress is a common complication in pregnant women. For gravid patients with comorbidities, the risk of acute respiratory distress in the presence of COVID-19 infection is even greater [10]. Due to the normal physiological adaptations of pregnancy, COVID-19 may further strain an already overworked respiratory system, making delivery one of the only viable options in helping a pregnant patient to recover [3,4]. Additionally, normal physiologic changes in pregnancy such as increased heart rate and oxygen consumption along with changes in cell-mediated immunity can further increase the risk of COVID in pregnant women [11]. Furthermore, new data show that infection with the SARS-CoV-2 virus can cause rare complications such as multisystem inflammatory syndrome in pregnant women [1].

Maternal and fetal status ultimately determine the timing and method of delivery in the critically ill patient in accordance with obstetrical guidelines. This patient was induced for vaginal delivery due to the viable fetal gestational age and the significantly decreased physiological stress of vaginal delivery as compared to operative delivery [12]. While delivery of a pregnant patient with respiratory failure has not been consistently shown to provide significant improvement in maternal respiratory failure, delivery was performed in an attempt to improve maternal oxygenation [12].

This case highlights the importance of not only treating COVID-19 in pregnant women but also infection prevention. Pregnant women should strive to mitigate exposure risk and be fully vaccinated against COVID-19. Clinical trials have demonstrated no serious adverse effects after vaccination in pregnant women [13]. The Center for Disease Control, the American College of Obstetrics and Gynecology, and the Society of Maternal Fetal Medicine recommend all pregnant women receive vaccination against the SARS-CoV-2 virus.

## Conclusions

The effects of SARS-CoV-2 virus during pregnancy have important implications and require further research. Current literature shows that infection with COVID-19 leads to increased maternal complications as compared to pregnant women without COVID-19 infection. COVID-19 infection has been shown to cause serious complications in pregnancy and the postpartum period, especially in patients that already have underlying risk factors. It is vital for pregnant women who are infected with COVID-19 to undergo careful monitoring for both maternal and fetal complications. Our case highlights how inducing preterm birth can provide maternal benefit for a woman experiencing respiratory complications of COVID-19. There is a need for more research detailing clinical progress and management of pregnancy complicated by COVID-19 infection.
